# Role of MIF Cytokine/CD74 Receptor Pathway in Protecting Against Injury and Promoting Repair

**DOI:** 10.3389/fimmu.2020.01273

**Published:** 2020-06-23

**Authors:** Laura Farr, Swagata Ghosh, Shannon Moonah

**Affiliations:** Department of Medicine, University of Virginia, Charlottesville, VA, United States

**Keywords:** wound healing, regeneration, cytokines, macrophage migration inhibitory factor (MIF), CD74 receptor, inflammatory bowel disease (IBD), ischemia-reperfusion injury (I/R), lung injury

## Abstract

Wound healing after an injury is essential for life. An in-depth understanding of the healing process is necessary to ultimately improve the currently limited treatment options for patients suffering as a result of damage to various organs and tissues. Injuries, even the most minor, trigger an inflammatory response that protects the host and activates repair pathways. In recent years, substantial progress has been made in delineating the mechanisms by which inflammatory cytokines and their receptors facilitate tissue repair and regeneration. This mini review focuses on emerging literature on the role of the cytokine macrophage migration inhibitory factor (MIF) and its cell membrane receptor CD74, in protecting against injury and promoting healing in different parts of the body.

## Introduction

Whenever an injury occurs, the body needs to repair it efficiently in order to protect from further damage and restore function. From minor scratches to myocardial infarction, we continually experience traumatic events throughout life. Therefore, the healing process is essential for survival. Further understanding of the mechanisms that promote healing could lead to new therapeutic opportunities to improve the lives of individuals with illnesses that resulted from organ and tissue injury (1, 2). In addition to protecting against invading pathogens, an appropriate inflammatory response activates repair pathways that are essential for healing, without causing unwanted damage to the host tissue. Cytokines play a crucial role in inflammation-driven repair. Cytokines act by binding to specific receptors on certain cell types triggering downstream signaling events that ultimately promote the healing process (3, 4).

**Figure 1:**
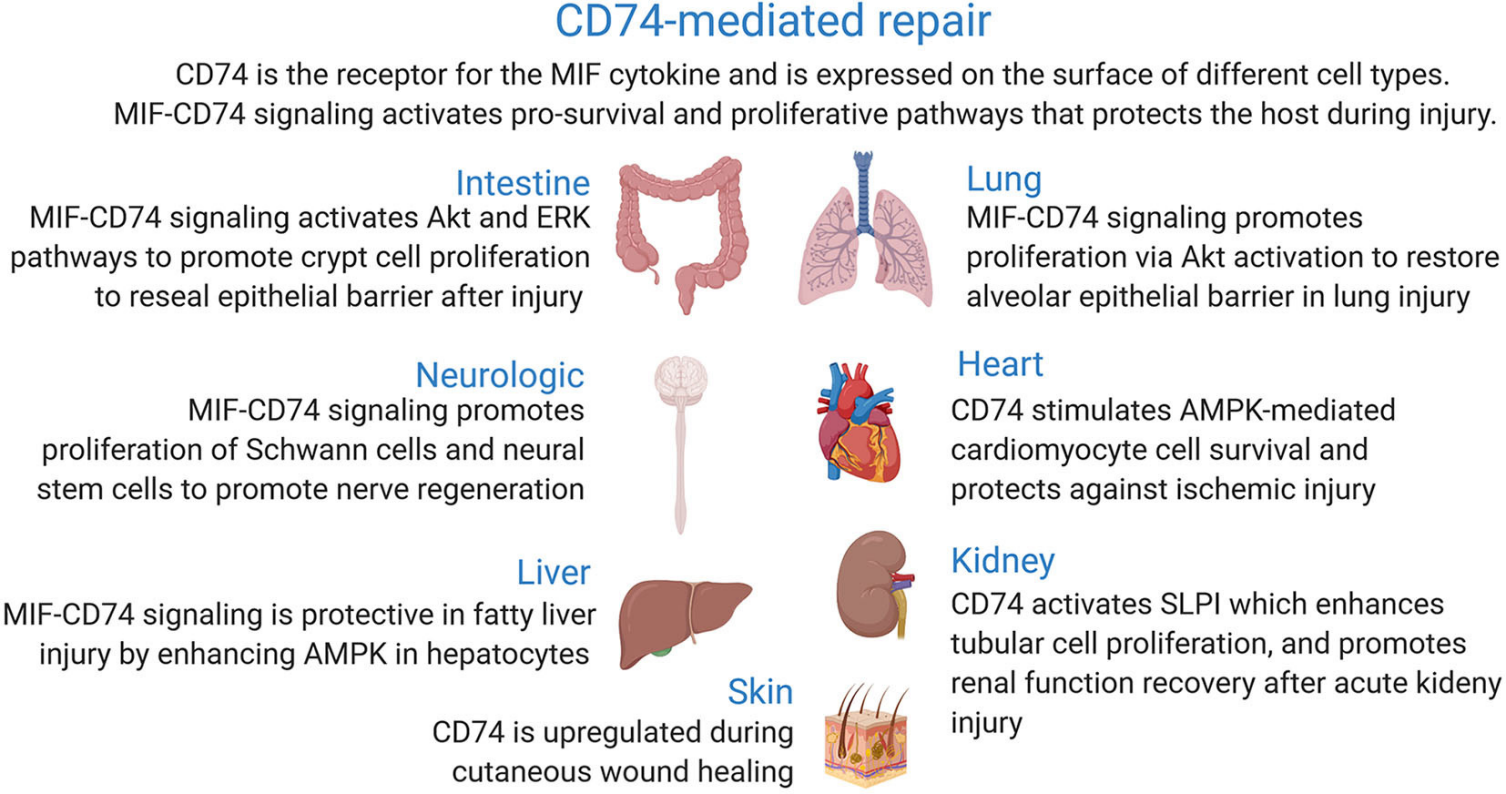
Role of CD74 receptor in tissue injury and wound repair.

This review focuses on the recent advances that have greatly contributed to our current understanding of the link between the signaling pathways activated upon binding of macrophage migration inhibitory factor cytokine to its membrane receptor CD74 and wound healing in different body parts (Figure 1).

## Macrophage Migration Inhibitory Factor

Macrophage migration inhibitory factor (MIF) is one of the first described cytokines, identified as a soluble immune cell-derived factor over 50 years ago in 1966. Similar to cytokines such as tumor necrosis factor (TNF), MIF's range of functions has exceeded what is implied by the historical name (5, 6). The MIF gene was cloned in 1989, and subsequent studies have demonstrated a wide range of roles for MIF. MIF is a truly pleiotropic inflammatory cytokine that is expressed by a variety of cells, and is a critical upstream mediator of innate immunity. Given its important role in immunity, it is not surprising that excess MIF expression has been linked to exaggerated inflammation and immunopathology. In addition, MIF demonstrates well-documented proliferative properties. MIF is secreted by many different types of cells and interacts with several receptors, which helps to explain the variety of biological functions. Receptors that interact that bind MIF include CD74, and chemokine receptors CXCR2 and CXCR4 (7–15).

## CD74

CD74 is a type II transmembrane protein consisting of an N-terminal cytosolic tail, a short transmembrane region, and a long C-terminus luminal region. Human CD74 is encoded on chromosome 5 and consists of four isoforms. Isoforms p33 and p41 are generated by alternative splicing, that is, the p33 isoform is created by excluding exon 6b from p41 CD74 transcript. Isoforms p35 and p43 originate from an alternative start site (16–21). While CD74 was first discovered in 1979 through co-immunoprecipitation of the Major Histocompatibility Class II antigen (MHCII), it wasn't until 1989 the antigen presentation function of CD74 was recognized. CD74 is expressed on classical antigen presenting cells (APCs), such as dendritic cells and macrophages, acts as a chaperone that binds MHCII, and is commonly referred to as the Class II invariant chain (Ii) (16, 18, 22, 23).

Subsequently, a growing body of evidence supported the concept that CD74 could have additional functions as a receptor. Surface expression of CD74 occurred independently of concomitant MHCII expression. Additionally, CD74 expression was found on the surface of non-APCs such as endothelial cells, and epithelial cells in the kidney, lung, gut, and skin (24, 25).

## CD74 Is A Receptor for MIF Cytokine

The receptor that mediated MIF activity remained elusive until a study in 2003, which utilized a cDNA library and fluorescently conjugated MIF to screen for a receptor and identified CD74 as the MIF receptor. The authors described that MIF bound to the extracellular domain of CD74, resulting in extracellular signal-regulated kinase (ERK) pathway activation (25). MIF-induced ERK activation through CD74 appears to depend on CD74 forming a complex with co-receptor CD44 (CD74/CD44) (26, 27). In addition to ERK, stimulation of CD74 has been shown to trigger activation of the PI3K-Akt signal transduction cascade, NF-κB, and the AMP-activated protein kinase (AMPK) pathways. These pathways play important roles in cell proliferation and survival (28).

D-dopachrome tautomerase (D-DT, MIF-2) was recently described as a member of the MIF protein superfamily, demonstrating overlapping inflammatory and proliferative properties with MIF. D-DT and MIF genes are located in close proximity on chromosome 22, ~80 kb apart. The amino acid sequence of human MIF and D-DT shows 34% identity, however, the structure of the two proteins is highly conserved. D-DT binds CD74 and initiates similar signaling pathways (29, 30). MIF homologs are also expressed by parasites. These MIF homologs are structurally and functionally similar to human MIF and interact with CD74. While it may seem counter-intuitive for protozoans to secrete MIF, parasite MIF appears to contribute to immune evasion and invasion (31–33).

## Regulation

Regulation of MIF-CD74 interactions occurs at several levels. MIF is constitutively expressed with increased MIF secretion occurring early in the inflammatory response. Triggers of increased MIF release include lipopolysaccharide (LPS) and cell injury. Secreted MIF then interacts with CD74 to carry out some if its functions (10, 14). CD74 activity is regulated by changes in expression, proteolytic processing, and MIF-interacting proteins that prevent binding to CD74. Similar to MIF, CD74 is expressed on multiple cells: immune cells (e.g., B lymphocytes, macrophages, dendritic cells) and non-immune cells including epithelial cells. Information on the regulation of CD74 expression in these different cells remains limited. Increased CD74 expression is observed in injury, inflammation, and cancer. IFN-γ, a cytokine crucial to both innate and adaptive immunity, increases CD74 expression in a variety of cells (34–36). Intracellular binding partners released in the extracellular space can regulate cytokine activity. Both ribosomal protein S19 (RPS19) and c-Jun activation domain binding protein 1 (JAB1) were shown to have regulatory effects by binding to MIF, inhibiting its interaction with CD74 (37, 38). CD74 also exists in a soluble CD74 ectodomain form which results from proteolytic shedding of the ectodomain region. However, the molecular mechanism including the protease responsible for releasing CD74 ectodomain remains poorly understood. Ectodomain shedding decreases the amount of CD74 surface receptors available to interact with MIF. Also, CD74 ectodomain regulates MIF activity by acting as a decoy receptor, sequestering free MIF to negatively regulate MIF signaling (39–41). Another proteolytic step involves signal peptide peptidase-like 2a (SPPL2a), which is an aspartic intramembrane protease. SPPL2a has shown to play an important role in CD74 proteolysis (42, 43). Yet, the exact role of SPPL2a-mediated CD74 proteolysis in MIF signaling and whether modulating SPPL2a enzyme activity affects MIF proinflammatory and proliferative functions remain to be fully investigated (16).

In the following sections, we summarize the recent data supporting the reparative role of MIF-CD74 signaling in different organs and tissues during injury. The role of CD74 in other disease processes, antigen presentation, and cancer has been well-reviewed elsewhere (16, 16–18, 28, 44–48).

## MIF-CD74 Signaling in Promoting Mucosal Healing During Colitis-Associated Injury

Inflammatory bowel disease (IBD), exemplified by Crohn's disease (CD) and ulcerative colitis (UC), is a growing public health challenge and socio-economic problem that affect millions with rapidly increasing incidence worldwide (49, 50). Mucosal healing has been established as an important treatment predictor of sustained clinical remission and resection-free survival in IBD (51). Unfortunately, a significant number of IBD patients do not respond to current treatment (including corticosteroids or biologics), and as many as 70% of CD and 25% of UC patients require surgical resection of affected regions of their intestine (52). Current therapeutic strategies focus on limiting inflammation, thus, there is an urgent need to develop new approaches that also facilitate tissue repair and mucosal healing.

Our understanding of the genetic contributions to IBD has seen significant advances over the past few decades. Genome-wide association studies (GWAS) have identified new single nucleotide polymorphisms (SNPs) associated with IBD predisposition and treatment failure (53, 54). A recent study aimed at determining genetic factors associated with poor response to anti-TNF therapy, found that a strong association between a CD74 polymorphism and anti-TNF failure in patients with ulcerative colitis. The rs7709772 SNP is located in the CD74 promoter region. The odds ratio for non-response to anti-TNF therapy with this SNP was relatively high at 22 (55).

**Figure 2:**
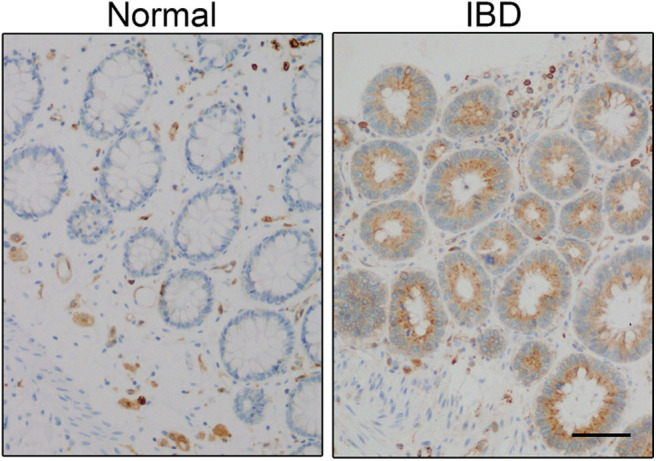
CD74 expression is increased in inflammatory bowel disease (IBD). CD74 (brown) is increased in significant amounts in proliferating crypt epithelial cells in the gut of IBD patient. Scale bar: 50 μm. Panel is reproduced from Farr et al. (58) with permission.

CD74 gene expression is increased in patients with IBD (56, 57), which occurs in the inflamed areas compared with non-inflamed and healthy intestine (Figure 2). CD74 overexpression was most noticeable in proliferating crypt epithelial cells of patients with IBD and amebic colitis, a condition often misdiagnosed as IBD (58, 59). CD74 is almost undetectable in the epithelium of non-inflamed human and mice intestine when analyzed by immunohistochemistry (58, 60, 61). Therefore, it was not too surprising to find that CD74 deficient mice had normal colon, histology, and barrier integrity, and lacked spontaneous colitis in the absence of pathologic insults (58). On the other hand, MIF is expressed by epithelial cells that line the intestine and MIF-deficient mice have impaired intestinal barrier integrity (62). Using a combination of genetic knock-out, bone marrow chimera mice, chemical, non–chemically-induced, acute, and chronic mouse models of colitis, CD74 was found to be essential for mucosal healing in colitis-associated injury. At the cellular level, MIF stimulation of CD74 on intestinal epithelial cells increased cell proliferation and wound closure, an effect that was lost in CD74-deficient cells. Mechanistically, MIF, which also is increased in colitis, stimulated the CD74 receptor, activating pro-proliferative Akt and ERK pathways (58). So while dispensable in steady state conditions, CD74 appears to be necessary for reparative inflammation.

Based on these findings, enhancing the CD74 pathway might represent a unique treatment approach for promoting healing in IBD. Though, finding the right ligand to stimulate CD74 may present a challenge. That is, stimulation of CD74 with exogenous MIF might lead to an excessive inflammatory state, as MIF is capable of stimulating CXCR2 and CXCR4 receptors in addition to CD74. CXCR2 and CXCR4 receptors when activated promote influx of neutrophils and lymphocytes, respectively (63, 64).

## MIF-CD74 Pathway in Recovery From Lung Injury

Lung injury arises from a wide variety of insults, which include pulmonary infections, such as bacterial and viral pneumonia caused by influenza and coronavirus, vaping-associated pulmonary illness (VAPI), ischemia–reperfusion-induced lung injury, and ventilator-induced lung injury (65–67). In the 2018–2019 season, influenza caused around 500,000 hospitalizations and 34,000 deaths (68). The emerging CoVID-19 has an increasing impact through infections and deaths as well as the economic impacts of quarantines and event cancellations to reduce infection spread (69, 70).

Lung injury causes damage to the epithelium. The alveolar epithelial barrier consists of two main cell types: alveolar epithelial type I and type II cells. Type I cells are flat cells through which gas exchange takes place and occupies most of the alveolar surface area. Type II cells serve as progenitor cells for the alveolar epithelium. Type I cells are more sensitive to injury and are predominantly destroyed during lung damage. Type II cells proliferate and differentiate into type I cells, thus actively reforming the alveolar epithelium after damage and promoting alveolar repair (71). Type II cells express CD74 on their surface. During acute injury such as viral infection, type I cells release MIF. Extracellular MIF binds to CD74 on adjacent type II epithelial cells, activating Akt and ERK pathways, resulting in cell proliferation and differentiation to restore the alveolar barrier (72).

Lung endothelial cells display almost undetectable amounts of CD74 at baseline. A recent study found that chronic hyperoxia led to CD74 upregulation in endothelial cells (73). Hyperoxia is common in patients with adult respiratory distress syndrome (ARDS), which is due to the requirement for high levels of supplemental oxygen. Endothelial injury is a key feature of hyperoxic acute lung injury (74). MIF-CD74 activation was found to protect from oxidative stress in an animal model. MIF and CD74 genetic knock-outs, and pharmacological inhibition of CD74 resulted in loss of the protective effects of CD74. This led to increases in inflammatory cytokines, apoptosis, and mortality. At the molecular level, CD74 activation during hyperoxia induced proliferative and pro-survival effects through ERK and Akt activation (73).

Neutrophils appear to play a significant role in tissue damage and the development of acute lung injury (75). It is important to mention that excess MIF was shown to correlate with neutrophil accumulation into the lung (76). However, it remains unclear how much MIF-CXCR2 interaction is contributing to leukocyte recruitment.

## MIF-CD74 Pathway in Recovery From Kidney Injury

Acute kidney injury (AKI) remains a significant medical problem and is associated with increased hospital mortality, length of stay, and costs. Individuals who survive an AKI hospitalization are likely to fail renal function recovery and go on to develop chronic kidney disease and hypertension (77). Most cases of AKI are due to ischemia, but our kidneys are also vulnerable to damage by toxins, infection, and immune-mediated insults. Ischemic AKI, for example, results in significant renal tubular cell damage. Free radicals formed during ischemia and reperfusion (I/R) also contribute to renal damage. Surviving cells undergo epithelium regeneration to restore healthy renal function (78, 79). A better understanding of the repair processes underlying kidney repair will facilitate therapies that will prevent injury, promote recovery, and minimize the progression to chronic kidney disease.

CD74 is expressed on the surface of renal tubular epithelial cells. Also, these cells express low levels of MIF which is increased following AKI to ensure adequate supplies at the site of damage (80, 81). A spontaneous pathological renal phenotype is absent MIF knock-out mice, suggesting little to no effect on healthy organs (82). However, high MIF levels can be found in the serum of patients following cardiac surgery and correlates with protection from AKI (81). In a murine model of experimental ischemia-reperfusion injury, MIF, MIF-2, and CD74 knock-out mice had worse tubular injury compared to wild type control mice. MIF-2 improved the recovery of injured epithelial cells by enhancing cell regeneration through secretory leukocyte proteinase inhibitor (SLPI) and activating transcription factor (ATF) 4-dependent mechanisms (83). SLPI has proliferative, antioxidant and cytoprotective properties, and is being evaluated as a biomarker for AKI after surgery (84–86).

While MIF/MIF-2 are likely protective in IR, this might not be the case for all renal diseases depending on the underlying pathology. For example, MIF has been linked to injury and inflammation in models of glomerular diseases (87–89). Therefore, additional studies are required to determine which patient conditions would benefit from blockade vs. stimulation strategies.

## CD74 Signaling in Protecting the Heart After Injury

Cardiovascular disease is the leading cause of death in the United States. Risk factors for cardiovascular disease include smoking, obesity, and hypertension. Myocardial infarction, or heart attack, occurs in one American every 40 s (90). Treatment for MI is composed of anti-coagulant medication, thrombolytics, and surgical intervention to restore normal blood flow. However, damage to cardiomyocytes caused by ischemia is not addressed in the standard treatment regimen and can lead to heart failure. Targeting repair of heart tissue during MI may improve patient outcomes and prevent chronic disease.

CD74 signaling was shown to have protective effects in cardiomyocytes in cardiac I/R injury animal model. MIF is secreted from the cardiomyocytes during I/R and acts in an autocrine-paracrine manner, stimulating cell surface CD74 receptor. Activation of CD74 with exogenous MIF-2 improved cell survival and infarct size both in wild-type control and conditional MIF-2 knockout mice, while CD74 deletion led to worse injury. Mechanistically, MIF-2 binding to CD74 quickly activates the AMP-activated protein kinase (AMPK) cascade via a calcium dependent kinase, CaMMK2 (91). Activation of the AMPK pathway in cardiomyocytes decreases apoptosis, necrosis, and contractile dysfunction following ischemia (91, 92). MIF-2 in contrast to MIF appears to lack the necessary CXCR-interacting motifs necessary for activation, and it is believed to exert a more selective action in activating the tissue-protective CD74 signaling pathway. That said, MIF triggers the CD74/CD44/AMPK receptor signaling pathway, which promotes glucose uptake in cardiomyocytes and protects the heart during ischemia-reperfusion injury (93, 94). Further studies are required to determine the potential of MIF/MIF-2 as a treatment strategy to protect the heart against ischemic injury.

## CD74 and Cutaneous Wound Healing

Impaired wound healing in the setting of non-healing surgical or traumatic wounds, pressure ulcers, diabetic foot ulcers, venous, and ischemic ulcers, presents a substantial healthcare burden. Chronic non-healing wounds contribute to significant healthcare costs, poor quality of life, and serious outcomes such as amputations (95, 96).

Following injury, several cytokines play important roles during tissue repair and promote cutaneous wound healing by the classic stages of wound repair: inflammation, new tissue formation, and remodeling (97, 98). Therefore, cytokine pathways have been targeted when designing regenerative strategies to promote chronic wound repair (99). Gene expression studies have been valuable for identifying cytokines expressed during the inflammatory process in a wound setting (100). A study analyzing gene expression profiles in patients with punch biopsies found MIF gene expression increased during cutaneous wound healing (101). The role of MIF in promoting wound healing was investigated using an animal model of skin injury. MIF levels were elevated early after injury and facilitated proliferation and migration of keratinocytes from the edge of the wound (102). These results support a reparative response of MIF to cutaneous injury. In addition, transcriptomic analysis revealed CD74 upregulated in pressure ulcers in a neuropathic ulcer mouse model (103). It is plausible that the MIF-CD74 pathway promotes cutaneous wound repair, however, further studies will be required to characterize the role of CD74 signaling in cutaneous wound healing.

## CD74 Activity in Other Organs and Tissues

CD74 signaling has also been found to play a potential role in healing in other tissues such as the nervous system and liver. Sciatica is a chronically painful disease caused by injury to the sciatic nerve. Schwann cells express CD74, and MIF is upregulated following sciatic nerve injury. MIF-stimulated CD74 activation of the ERK pathway led to Schwann cell proliferation and subsequent nerve regeneration. Also, *in vitro* studies show that MIF facilitates Schwann cell migration. Both Schwann cell proliferation and migration promote nerve regeneration (104). A separate *in vitro* study demonstrated that CD74 activation by MIF promoted cell survival and proliferation of neural progenitor cells (105). Further studies will be required to determine if MIF-induced proliferation of neural progenitor cells can be a therapeutic option in brain disorders. In the liver, CD74-MIF signaling plays a protective role in nonalcoholic fatty liver disease (NAFLD) by enhancing AMPK (106).

## Proinflammatory Effects and Disease Outcomes Linked to MIF-CD74 Signaling

While this review focuses on the protective role of MIF-CD74 signaling, it should be noted that this is not the case for all diseases (18, 44, 107). The complex pathological processes that result in disease combined with CD74's expression on a variety of cell types, and its multiple co-receptors with diverse downstream signaling pathways contribute to these varied outcomes. For example, lupus nephritis is inflammation of the kidney that is caused by the autoimmune disease systemic lupus erythematosus (SLE) (108). B cells participate in SLE immunopathogenesis (109). B lymphocytes express elevated levels of CD74 in mouse models of SLE and lupus-prone mouse strains have elevated MIF. Both MIF and CD74 elevated expression positively correlated with worsening inflammation. MIF inhibition and CD74 deficiency protected against glomerulonephritis in lupus-prone mice (110, 111). Despite these results that suggest MIF-CD74 pathway plays a role in lupus pathology, a phase 1 clinical trial of an anti-MIF monoclonal antibody in lupus nephritis was terminated early for unclear reasons (112). These findings suggest that MIF-CD74 functions with differential outcomes occur in a context- and cell type-dependent manner. Given this complexity, additional research is needed to determine when and how to inhibit or stimulate the MIF-CD74 pathway to achieve benefit. Also, whether disease associations are a result of different co-receptor involvement on different cell types should be a focus of future research.

MIF's proinflammatory effects involve enhancing the expression of various cytokines such as TNF-α, IL-6, IL-8 (14). Cytokines like IL-6 are now recognized for their roles triggering tissue repair and regeneration (4, 113). While these downstream proinflammatory MIF effects have been linked to immune disorders, it remains possible that they play a role in the healing effects of MIF-CD74 signaling. This would be an interesting area for future investigation as balancing the positive and negative effects of MIF appears to be key.

## Conclusion

Discussed above is the recurrent observations of the protective effects of MIF-CD74 signaling in wound-healing. Recent studies have furthered our understanding of the mechanisms by which CD74 stimulation leads to tissue repair in multiple parts of the body involving some of the most important diseases. Despite these advances, key questions remain unanswered. For example, although there is mechanistic overlap, the downstream pathways that are important for CD74-mediated repair appear to vary with the tissue or cell type. In epithelial cells, such as those that line the gut and alveoli of the lungs, MIF-CD74 interaction triggers the activation of pro-survival and proliferative Akt and ERK pathways. In contrast, activation of the pro-survival kinase AMPK seems to play a more significant role in cardiomyocytes and hepatocytes. The molecular reason for the different downstream signaling pathways beyond differences in cell types is not fully understood and present worthy unknowns to be solved by future studies. Furthermore, a selective agonist that will stimulate CD74-mediated repair with little or no unwanted side effects remains poorly defined. The answers to such questions may allow us to translate these recent scientific discoveries into clinical interventions, and ultimately benefit those suffering as a result of injury to various organs and tissues.

## Author Contributions

LF, SG, and SM wrote, edited and reviewed the manuscript. All authors contributed to the article and approved the submitted version.

## Conflict of Interest

The authors declare that the research was conducted in the absence of any commercial or financial relationships that could be construed as a potential conflict of interest.

## References

[1] Krafts KP. Tissue repair: the hidden drama. Organogenesis. (2010) 6:225–33. 10.4161/org.6.4.12555 21220961 PMC3055648

[2] Reinke J Sorg H. Wound repair and regeneration. Eur Surg Res. (2012) 49:35–43. 10.1159/000339613 22797712

[3] Eming SA Wynn TA Martin P. Inflammation and metabolism in tissue repair and regeneration. Science. (2017) 356:1026–30. 10.1126/science.aam7928 28596335

[4] Karin M Clevers H. Reparative inflammation takes charge of tissue regeneration. Nature. (2016) 529:307. 10.1038/nature17039 26791721 PMC5228603

[5] Bloom BR Bennett B. Mechanism of a reaction in vitro associated with delayed-type hypersensitivity. Science. (1966) 153:80–82. 10.1126/science.153.3731.80 5938421

[6] David JR. Delayed hypersensitivity in vitro: its mediation by cell-free substances formed by lymphoid cell-antigen interaction. Proc Natl Acad Sci USA. (1966) 56:72–7. 10.1073/pnas.56.1.72 5229858 PMC285677

[7] Bernhagen J Calandra T Mitchell R Martin S Tracey K Voelter W. MIF is a pituitary-derived cytokine that potentiates lethal endotoxaemia. Nature. (1993) 365:756–9. 10.1038/365756a0 8413654

[8] Weiser WY Temple PA Witek-Giannotti JS Remold HG Clark SC David JR. Molecular cloning of a cDNA encoding a human macrophage migration inhibitory factor. Proc Natl Acad Sci USA. (1989) 86:7522–6. 10.1073/pnas.86.19.7522 2552447 PMC298097

[9] Hudson JD Shoaibi MA Maestro R Carnero A Hannon GJ Beach DH. A proinflammatory cytokine inhibits p53 tumor suppressor activity. J Exp Med. (1999) 190:1375–82. 10.1084/jem.190.10.1375 10562313 PMC2195698

[10] Calandra T Roger T. Macrophage migration inhibitory factor: a regulator of innate immunity. Nat Rev Immunol. (2003) 3:791–800. 10.1038/nri1200 14502271 PMC7097468

[11] Lippitz BE. Cytokine patterns in patients with cancer: a systematic review. Lancet Oncol. (2013) 14:e218–e28. 10.1016/S1470-2045(12)70582-X 23639322

[12] Bucala R Donnelly SC. Macrophage migration inhibitory factor: a probable link between inflammation and cancer. Immunity. (2007) 26:281–5. 10.1016/j.immuni.2007.03.005 17376392

[13] Binsky I Haran M Starlets D Gore Y Lantner F Harpaz N. IL-8 secreted in a macrophage migration-inhibitory factor-and CD74-dependent manner regulates B cell chronic lymphocytic leukemia survival. Proc Natl Acad Sci USA. (2007) 104:13408–13. 10.1073/pnas.0701553104 17686984 PMC1948950

[14] Harris J VanPatten S Deen NS Al-Abed Y Morand EF. Rediscovering MIF: new tricks for an old cytokine. Trends Immunol. (2019) 40:447–62. 10.1016/j.it.2019.03.002 30962001

[15] Meyer-Siegler K Hudson PB. Enhanced expression of macrophage migration inhibitory factor in prostatic adenocarcinoma metastases. Urology. (1996) 48:448–52. 10.1016/S0090-4295(96)00207-5 8804500

[16] Schroder B. The multifaceted roles of the invariant chain CD74–More than just a chaperone. Biochim Biophys Acta. (2016) 1863:1269–81. 10.1016/j.bbamcr.2016.03.026 27033518

[17] Bucala R Shachar I. The integral role of CD74 in antigen presentation, MIF signal transduction, and B cell survival and homeostasis. Mini Rev Med Chem. (2014) 14:1132–8. 10.2174/1389557515666150203144111 25643611

[18] Becker-Herman S Gil N Radomir L Shachar I MIF- and CD74-Dependent, mechanisms. In: Bucala R Bernhagen J editors. MIF Family Cytokines in Innate Immunity and Homeostasis. Cham: Springer (2017). p. 1–20. 10.1007/978-3-319-52354-5_1

[19] Strubin M Berte C Mach B. Alternative splicing and alternative initiation of translation explain the four forms of the Ia antigen-associated invariant chain. EMBO J. (1986) 5:3483–8. 10.1002/j.1460-2075.1986.tb04673.x 3104027 PMC1167384

[20] Yamamoto K Koch N Steinmetz M Hämmerling G. One gene encodes two distinct Ia-associated invariant chains. J Immunol. (1985) 134:3461–7. 3920321

[21] Koch N Lauer W Habicht J Dobberstein B. Primary structure of the gene for the murine Ia antigen-associated invariant chains (Ii). An alternatively spliced exon encodes a cysteine-rich domain highly homologous to a repetitive sequence of thyroglobulin. EMBO J. (1987) 6:1677–83. 10.1002/j.1460-2075.1987.tb02417.x 3038530 PMC553541

[22] Jones PP Murphy DB Hewgill D McDevitt HO. Detection of a common polypeptide chain in IA and IE sub-region immunoprecipitates. Mol Immunol. (1979) 16:51–60. 10.1016/0161-5890(79)90027-0 376435

[23] Stockinger B Pessara U Lin RH Habicht J Grez M Koch N. A role of la-associated invariant chains in antigen processing and pressentation. Cell. (1989) 56:683–9. 10.1016/0092-8674(89)90590-4 2917369

[24] Henne C Schwenk F Koch N Möller P. Surface expression of the invariant chain (CD74) is independent of concomitant expression of major histocompatibility complex class II antigens. Immunology. (1995) 84:177. 7750992 PMC1415095

[25] Leng L Metz CN Fang Y Xu J Donnelly S Baugh J. MIF signal transduction initiated by binding to CD74. J Exp Med. (2003) 197:1467–476. 10.1084/jem.20030286 12782713 PMC2193907

[26] Shi X Leng L Wang T Wang W Du X Li J. CD44 is the signaling component of the macrophage migration inhibitory factor-CD74 receptor complex. Immunity. (2006) 25:595–606. 10.1016/j.immuni.2006.08.020 17045821 PMC3707630

[27] Naujokas MF Morin M Anderson MS Peterson M Miller J. The chondroitin sulfate form of invariant chain can enhance stimulation of T cell responses through interaction with CD44. Cell. (1993) 74:257–68. 10.1016/0092-8674(93)90417-O 8343954

[28] Su H Na N Zhang X Zhao Y. The biological function and significance of CD74 in immune diseases. Inflamm Res. (2017) 66:209–16. 10.1007/s00011-016-0995-1 27752708

[29] Merk M Zierow S Leng L Das R Du X Schulte W. The D-dopachrome tautomerase (DDT) gene product is a cytokine and functional homolog of macrophage migration inhibitory factor (MIF). Proc Natl Acad Sci USA. (2011) 108:E577–E85. 10.1073/pnas.1102941108 21817065 PMC3161582

[30] Merk M Mitchell RA Endres S Bucala R. D-dopachrome tautomerase (D-DT or MIF-2): doubling the MIF cytokine family. Cytokine. (2012) 59:10–7. 10.1016/j.cyto.2012.03.014 22507380 PMC3367028

[31] Ghosh S Jiang N Farr L Ngobeni R Moonah S. Parasite-Produced MIF cytokine: role in immune evasion, invasion, and pathogenesis. Front Immunol. (2019) 10:1995. 10.3389/fimmu.2019.01995 31497025 PMC6712082

[32] Sparkes A De Baetselier P Roelants K De Trez C Magez S Van Ginderachter JA. The non-mammalian MIF superfamily. Immunobiology. (2017) 222:473–482. 10.1016/j.imbio.2016.10.006 27780588 PMC5293613

[33] Ghosh S Padalia J Ngobeni R Abendroth J Farr L Shirley D-A Targeting parasite-produced macrophage migration inhibitory factor as an antivirulence strategy with antibiotic-antibody combination to reduce tissue damage. J Infect Dis. (2019) 20:1–9. 10.1093/infdis/jiz579 PMC7325720 31677380

[34] Tanese K Hashimoto Y Berkova Z Wang Y Samaniego F Lee JE. Cell surface CD74-MIF interactions drive melanoma survival in response to interferon-γ. J Invest Dermatol. (2015) 135:2775–84. 10.1038/jid.2015.204 26039541 PMC4640965

[35] Kursunel MA Esendagli G. The untold story of IFN-γ in cancer biology. Cytok Growth Factor Rev. (2016) 31:73–81. 10.1016/j.cytogfr.2016.07.005 27502919

[36] Guda MR Rashid MA Asuthkar S Jalasutram A Caniglia JL Tsung AJ. Pleiotropic role of macrophage migration inhibitory factor in cancer. Am J Cancer Res. (2019) 9:2760. 31911860 PMC6943360

[37] Filip AM Klug J Cayli S Frohlich S Henke T Lacher P. Ribosomal protein S19 interacts with macrophage migration inhibitory factor and attenuates its pro-inflammatory function. J Biol Chem. (2009) 284:7977–85. 10.1074/jbc.M808620200 19155217 PMC2658091

[38] Ghosh S Leaton LA Farr L Barfield A Moonah S. Interaction between parasite-encoded JAB1/CSN5 and macrophage migration inhibitory factor proteins attenuates its proinflammatory function. Sci Rep. (2018) 8:1. 10.1038/s41598-018-28625-1 29980718 PMC6035221

[39] Becker-Herman S Arie G Medvedovsky H Kerem A Shachar I. CD74 is a member of the regulated intramembrane proteolysis-processed protein family. Mol Biol Cell. (2005) 16:5061–69. 10.1091/mbc.e05-04-0327 16107560 PMC1266406

[40] Stoppe C Rex S Goetzenich A Kraemer S Emontzpohl C Soppert J. Interaction of MIF family proteins in myocardial ischemia/reperfusion damage and their influence on clinical outcome of cardiac surgery patients. Antioxid Redox Signal. (2015) 23:865–79. 10.1089/ars.2014.6243 26234719 PMC4615780

[41] Assis DN Leng L Du X Zhang CK Grieb G Merk M. The role of macrophage migration inhibitory factor in autoimmune liver disease. Hepatology. (2014) 59:580–91. 10.1002/hep.26664 23913513 PMC3877200

[42] Schneppenheim J Dressel R Hüttl S Lüllmann-Rauch R Engelke M Dittmann K. The intramembrane protease SPPL2a promotes B cell development and controls endosomal traffic by cleavage of the invariant chain. J Exp Med. (2013) 210:41–58. 10.1084/jem.20121069 23267015 PMC3549707

[43] Hüttl S Helfrich F Mentrup T Held S Fukumori A Steiner H. Substrate determinants of signal peptide peptidase-like 2a (SPPL2a)-mediated intramembrane proteolysis of the invariant chain CD74. Biochem J. (2016) 473:1405–22. 10.1042/BCJ20160156 26987812

[44] Valiño-Rivas L Baeza-Bermejillo C Gonzalez-Lafuente L Sanz AB Ortiz A Sanchez-Niño MD. CD74 in kidney disease. Front Immunol. (2015) 6:483. 10.3389/fimmu.2015.00483 26441987 PMC4585214

[45] Borghese F Clanchy FI. CD74: an emerging opportunity as a therapeutic target in cancer and autoimmune disease. Exp Opin Ther Targets. (2011) 15:237–51. 10.1517/14728222.2011.550879 21208136

[46] Beswick EJ Reyes VE. CD74 in antigen presentation, inflammation, and cancers of the gastrointestinal tract. W J Gastroenterol. (2009) 15:2855–61. 10.3748/wjg.15.2855 19533806 PMC2699002

[47] Fernandez-Cuesta L Plenker D Osada H Sun R Menon R Leenders F. CD74-NRG1 fusions in lung adenocarcinoma. Cancer Dis. (2014) 4:415–22. 10.1158/2159-8290.CD-13-0633 24469108

[48] Stein R Mattes MJ Cardillo TM Hansen HJ Chang C-H Burton J. CD74: a new candidate target for the immunotherapy of B-cell neoplasms. Clin Cancer Res. (2007) 13:5556s−63s. 10.1158/1078-0432.CCR-07-1167 17875789

[49] Ng SC Shi HY Hamidi N Underwood FE Tang W Benchimol EI. Worldwide incidence and prevalence of inflammatory bowel disease in the 21st century: a systematic review of population-based studies. Lancet. (2017) 390:2769–78. 10.1016/S0140-6736(17)32448-0 29050646

[50] Alatab S Sepanlou SG Ikuta K Vahedi H Bisignano C Safiri S. The global, regional, and national burden of inflammatory bowel disease in 195 countries and territories, 1990-2017: a systematic analysis for the Global Burden of Disease Study 2017. Lancet Gastroenterol Hepatol. (2020) 5:17–30. 10.1016/S2468-1253(19)30333-4 31648971 PMC7026709

[51] Neurath MF. Current and emerging therapeutic targets for IBD. Nat Rev Gastroenterol Hepatol. (2017) 14:269. 10.1038/nrgastro.2016.208 28144028

[52] Krezalek MA Cannon LM Hurst RD Update on the surgical treatment of inflammatory bowel disease. In: Cohen RD editor. Inflammatory Bowel Disease: Diagnosis and Therapeutics. Cham: Springer International Publishing (2017). p. 289–310. 10.1007/978-3-319-53763-4_17

[53] Loddo I Romano C. Inflammatory bowel disease: genetics, epigenetics, and pathogenesis. Front Immunol. (2015) 6:551. 10.3389/fimmu.2015.00551 26579126 PMC4629465

[54] McGovern DP Kugathasan S Cho JH. Genetics of inflammatory bowel diseases. Gastroenterology. (2015) 149:1163–76. e2. 10.1053/j.gastro.2015.08.001 26255561 PMC4915781

[55] Yoon SM Haritunians T Chhina S Liu Z Yang S Landers C. Colonic phenotypes are associated with poorer response to anti-TNF therapies in patients with IBD. Inflamm Bowel Dis. (2017) 23:1382–93. 10.1097/MIB.0000000000001150 28590340 PMC6510223

[56] Lawrance IC Fiocchi C Chakravarti S. Ulcerative colitis and Crohn's disease: distinctive gene expression profiles and novel susceptibility candidate genes. Hum Mol Genet. (2001) 10:445–56. 10.1093/hmg/10.5.445 11181568

[57] Parikh K Antanaviciute A Fawkner-Corbett D Jagielowicz M Aulicino A Lagerholm C. Colonic epithelial cell diversity in health and inflammatory bowel disease. Nature. (2019) 567:49–55. 10.1038/s41586-019-0992-y 30814735

[58] Farr L Ghosh S Jiang N Watanabe K Parlak M Bucala R. CD74 signaling links inflammation to intestinal epithelial cell regeneration and promotes mucosal healing. Cell Mol Gastroenterol Hepatol. (2020) 10:101–12. 10.1016/j.jcmgh.2020.01.009 32004754 PMC7215244

[59] Shirley D-A Moonah S. Fulminant amebic colitis after corticosteroid therapy: a systematic review. PLoS Negl Trop Dis. (2016) 10:e0004879. 10.1371/journal.pntd.0004879 27467600 PMC4965027

[60] Jiang Z Xu M Savas L LeClair P Banner B. Invariant chain expression in colon neoplasms. Virchows Archiv. (1999) 435:32–36. 10.1007/s004280050391 10431843

[61] Möller P Momburg F Koretz K Moldenhauer G Herfarth C Otto HF. Influence of major histocompatibility complex class I and II antigens on survival in colorectal carcinoma. Cancer research. (1991) 51:729–36. 1985791

[62] Vujicic M Saksida T Despotovic S Bajic SS Lalić I Koprivica I. The role of macrophage migration inhibitory factor in the function of intestinal barrier. Sci Rep. (2018) 8:1–2. 10.1038/s41598-018-24706-3 29679061 PMC5910418

[63] Bernhagen J Krohn R Lue H Gregory JL Zernecke A Koenen RR. MIF is a noncognate ligand of CXC chemokine receptors in inflammatory and atherogenic cell recruitment. Nat Med. (2007) 13:587. 10.1038/nm1567 17435771

[64] Weber C Kraemer S Drechsler M Lue H Koenen RR Kapurniotu A. Structural determinants of MIF functions in CXCR2-mediated inflammatory and atherogenic leukocyte recruitment. Proc Natl Acad Sci USA. (2008) 105:16278–83. 10.1073/pnas.0804017105 18852457 PMC2566990

[65] Butt YM Smith ML Tazelaar HD Vaszar LT Swanson KL Cecchini MJ. Pathology of vaping-associated lung injury. N Engl J Med. (2019) 381:1780–1. 10.1056/NEJMc1913069 31577870

[66] de Perrot M Liu M Waddell TK Keshavjee S. Ischemia-reperfusion-induced lung injury. Am J Resp Crit Care Med. (2003) 167:490–511. 10.1164/rccm.200207-670SO 12588712

[67] Slutsky AS Ranieri VM. Ventilator-induced lung injury. N Engl J Med. (2013) 369:2126–36. 10.1056/NEJMra1208707 24283226

[68] Rolfes MA Foppa IM Garg S Flannery B Brammer L Singleton JA. Annual estimates of the burden of seasonal influenza in the United States: a tool for strengthening influenza surveillance and preparedness. Influenza Other Resp Viruses. (2018) 12:132–7. 10.1111/irv.12486 29446233 PMC5818346

[69] Li Q Guan X Wu P Wang X Zhou L Tong Y. Early transmission dynamics in Wuhan, China, of novel coronavirus-infected pneumonia. N Engl J Med. (2020) 382:1199–207. 10.1056/NEJMoa2001316 31995857 PMC7121484

[70] Holshue ML DeBolt C Lindquist S Lofy KH Wiesman J Bruce H. First case of 2019 novel coronavirus in the United States. N Engl J Med. (2020) 382:929–36. 10.1056/NEJMoa2001191 32004427 PMC7092802

[71] Mason RJ. Biology of alveolar type II cells. Respirology. (2006) 11:S12–S5. 10.1111/j.1440-1843.2006.00800.x 16423262

[72] Marsh LM Cakarova L Kwapiszewska G von Wulffen W Herold S Seeger W. Surface expression of CD74 by type II alveolar epithelial cells: a potential mechanism for macrophage migration inhibitory factor-induced epithelial repair. Am J Physiol Lung Cell Mol Physiol. (2009) 296:L442–52. 10.1152/ajplung.00525.2007 19136583

[73] Sauler M Zhang Y Min JN Leng L Shan P Roberts S. Endothelial CD74 mediates macrophage migration inhibitory factor protection in hyperoxic lung injury. Faseb J. (2015) 29:1940–9. 10.1096/fj.14-260299 25609432 PMC4415022

[74] Kallet RH Matthay MA. Hyperoxic acute lung injury. Respir Care. (2013) 58:123–41. 10.4187/respcare.01963 23271823 PMC3915523

[75] Kolaczkowska E Kubes P. Neutrophil recruitment and function in health and inflammation. Nat Rev Immunol. (2013) 13:159–75. 10.1038/nri3399 23435331

[76] Takahashi K Koga K Linge HM Zhang Y Lin X Metz CN. Macrophage CD74 contributes to MIF-induced pulmonary inflammation. Resp Res. (2009) 10:33. 10.1186/1465-9921-10-33 19413900 PMC2681459

[77] Pavkov ME Harding JL Burrows NR. Trends in hospitalizations for acute kidney injury - united states, 2000-2014. MMWR Morb Mortal Wkly Rep. (2018) 67:289–93. 10.15585/mmwr.mm6710a2 29543788 PMC5857198

[78] Bonventre JV Yang L. Cellular pathophysiology of ischemic acute kidney injury. J Clin Invest. (2011) 121:4210–21. 10.1172/JCI45161 22045571 PMC3204829

[79] Devarajan P. Update on mechanisms of schemic acute kidney injury. J Am Soc Nephrol. (2006) 17:1503–20. 10.1681/ASN.2006010017 16707563

[80] Shachar I. An essential MIF-CD74 signaling axis in kidney tubular regeneration, with prospects for precision medicine and pharmacological augmentation. Am J Physiol Renal Physiol. (2017) 2017:F1084–6. 10.1152/ajprenal.00283.2017 28615250

[81] Stoppe C Averdunk L Goetzenich A Soppert J Marlier A Kraemer S. The protective role of macrophage migration inhibitory factor in acute kidney injury after cardiac surgery. Sci Transl Med. (2018) 10:4886. 10.1126/scitranslmed.aan4886 29769287

[82] Djudjaj S Martin IV Buhl EM Nothofer NJ Leng L Piecychna M. Macrophage migration inhibitory factor limits renal inflammation and fibrosis by counteracting tubular cell cycle arrest. J Am Soc Nephrol. (2017) 28:3590–604. 10.1681/ASN.2017020190 28801314 PMC5698074

[83] Ochi A Chen D Schulte W Leng L Moeckel N Piecychna M. MIF-2/D-DT enhances proximal tubular cell regeneration through SLPI- and ATF4-dependent mechanisms. Am J Physiol Renal Physiol. (2017) 313:F767–80. 10.1152/ajprenal.00683.2016 28539339 PMC6148305

[84] Averdunk L Rückbeil MV Zarbock A Martin L Marx G Jalaie H. SLPI-a biomarker of acute kidney injury after open and endovascular thoracoabdominal aortic aneurysm (TAAA) repair. Sci Rep. (2020) 10:9. 10.1038/s41598-020-60482-9 32103084 PMC7044192

[85] Averdunk L Fitzner C Levkovich T Leaf DE Sobotta M Vieten J. Secretory leukocyte protease inhibitor (SLPI)-A novel predictive biomarker of acute kidney injury after cardiac surgery: a prospective observational study. J Clin Med. (2019) 8:1931. 10.3390/jcm8111931 31717603 PMC6912354

[86] Zhang D Simmen RC Michel FJ Zhao G Vale-Cruz D Simmen FA. Secretory leukocyte protease inhibitor mediates proliferation of human endometrial epithelial cells by positive and negative regulation of growth-associated genes. J Biol Chem. (2002) 277:29999–30009. 10.1074/jbc.M203503200 12023969

[87] Leng L Chen L Fan J Greven D Arjona A Du X. A small-molecule macrophage migration inhibitory factor antagonist protects against glomerulonephritis in lupus-prone NZB/NZW F1 and MRL/lpr mice. J Immunol. (2011) 186:527–38. 10.4049/jimmunol.1001767 21106847 PMC3124407

[88] Hoi AY Hickey MJ Hall P Yamana J O'Sullivan KM Santos LL. Macrophage migration inhibitory factor deficiency attenuates macrophage recruitment, glomerulonephritis, and lethality in MRL/lpr mice. J Immunol. (2006) 177:5687–96. 10.4049/jimmunol.177.8.5687 17015758

[89] Leung JC Chan LY Tsang AW Liu EW Lam MF Tang SC. Anti-macrophage migration inhibitory factor reduces transforming growth factor-β1 expression in experimental IgA nephropathy. Nephrol Dial Transpl. (2004) 19:1976–85. 10.1093/ndt/gfh323 15187193

[90] Benjamin EJ Muntner P Alonso A Bittencourt MS Callaway CW Carson AP. Heart disease and stroke statistics-2019 update: a report from the american heart association. Circulation. (2019) 139:e56–e28. 10.1161/CIR.0000000000000659 30700139

[91] Qi D Atsina K Qu L Hu X Wu X Xu B. The vestigial enzyme D-dopachrome tautomerase protects the heart against ischemic injury. J Clin Invest. (2014) 124:3540–50. 10.1172/JCI73061 24983315 PMC4109524

[92] Qi D Young LH. AMPK: energy sensor and survival mechanism in the ischemic heart. Trends Endocrinol Metab. (2015) 26:422–9. 10.1016/j.tem.2015.05.010 26160707 PMC4697457

[93] Miller EJ Li J Leng L McDonald C Atsumi T Bucala R. Macrophage migration inhibitory factor stimulates AMP-activated protein kinase in the ischaemic heart. Nature. (2008) 451:578–82. 10.1038/nature06504 18235500

[94] Liu X Li X Zhu W Zhang Y Hong Y Liang X. Exosomes from mesenchymal stem cells overexpressing MIF enhance myocardial repair. J Cell Physiol. (2020). 10.1002/jcp.29456. [Epub ahead of print]. 31960418

[95] Sen CK Gordillo GM Roy S Kirsner R Lambert L Hunt TK. Human skin wounds: a major and snowballing threat to public health and the economy. Wound Repair Regen. (2009) 17:763–71. 10.1111/j.1524-475X.2009.00543.x 19903300 PMC2810192

[96] Järbrink K Ni G Sönnergren H Schmidtchen A Pang C Bajpai R. The humanistic and economic burden of chronic wounds: a protocol for a systematic review. Syst Rev. (2017) 6:15. 10.1186/s13643-016-0400-8 28118847 PMC5259833

[97] Werner S Grose R. Regulation of wound healing by growth factors and cytokines. Physiol Rev. (2003) 83:835–70. 10.1152/physrev.2003.83.3.835 12843410

[98] Lau K Paus R Tiede S Day P Bayat A. Exploring the role of stem cells in cutaneous wound healing. Exp Dermatol. (2009) 18:921–33. 10.1111/j.1600-0625.2009.00942.x 19719838

[99] Larouche J Sheoran S Maruyama K Martino MM. Immune regulation of skin wound healing: mechanisms and novel therapeutic targets. Adv Wound Care. (2018) 7:209–31. 10.1089/wound.2017.0761 29984112 PMC6032665

[100] Shaw TJ Martin P. Wound repair at a glance. J Cell Sci. (2009) 122:3209–13. 10.1242/jcs.031187 19726630 PMC2736861

[101] Deonarine K Panelli MC Stashower ME Jin P Smith K Slade HB. Gene expression profiling of cutaneous wound healing. J Transl Med. (2007) 5:11. 10.1186/1479-5876-5-11 17313672 PMC1804259

[102] Abe R Shimizu T Ohkawara A Nishihira J. Enhancement of macrophage migration inhibitory factor (MIF) expression in injured epidermis and cultured fibroblasts. Mol Basis Dis. (2000) 1500:1–9. 10.1016/S0925-4439(99)00080-0 10564712

[103] Benčová S Transcriptomic analysis of cutaneous inflammatory biomarkers in a mouse model of small fiber neuropathy. (2018).

[104] Song H Zhu Z Zhou Y Du N Song T Liang H. MIF/CD74 axis participates in inflammatory activation of Schwann cells following sciatic nerve injury. J Mol Histol. (2019) 50:355–67. 10.1007/s10735-019-09832-0 31197516

[105] Ohta S Misawa A Fukaya R Inoue S Kanemura Y Okano H. Macrophage migration inhibitory factor (MIF) promotes cell survival and proliferation of neural stem/progenitor cells. J Cell Sci. (2012) 125:3210–20. 10.1242/jcs.102210 22454509

[106] Heinrichs D Berres ML Coeuru M Knauel M Nellen A Fischer P. Protective role of macrophage migration inhibitory factor in nonalcoholic steatohepatitis. Faseb J. (2014) 28:5136–47. 10.1096/fj.14-256776 25122558 PMC4232286

[107] Fagone P Mazzon E Cavalli E Bramanti A Petralia MC Mangano K. Contribution of the macrophage migration inhibitory factor superfamily of cytokines in the pathogenesis of preclinical and human multiple sclerosis: *In silico* and *in vivo* evidences. J Neuroimmunol. (2018) 322:46–56. 10.1016/j.jneuroim.2018.06.009 29935880

[108] Almaani S Meara A Rovin BH. Update on lupus nephritis. Clin J Am Soc Nephrol. (2017) 12:825–35. 10.2215/CJN.05780616 27821390 PMC5477208

[109] Dörner T Giesecke C Lipsky PE. Mechanisms of B cell autoimmunity in SLE. Arthri Res Ther. (2011) 13:243. 10.1186/ar3433 22078750 PMC3308063

[110] Zhou Y Chen H Liu L Yu X Sukhova GK Yang M. CD74 deficiency mitigates systemic lupus erythematosus-like autoimmunity and pathological findings in mice. J Immunol. (2017) 198:2568–77. 10.4049/jimmunol.1600028 28219888 PMC5360510

[111] Lapter S Ben-David H Sharabi A Zinger H Telerman A Gordin M. A role for the B-cell CD74/macrophage migration inhibitory factor pathway in the immunomodulation of systemic lupus erythematosus by a therapeutic tolerogenic peptide. Immunology. (2011) 132:87–95. 10.1111/j.1365-2567.2010.03342.x 20738420 PMC3015078

[112] Corporation BH Anti-Macrophage migration inhibitory factor (anti-mif) antibody in lupus nephritis. ClinicalTrials.gov NCT (NCT01541670). (2012).

[113] Taniguchi K Wu L-W Grivennikov SI De Jong PR Lian I Yu F-X. A gp130-Src-YAP module links inflammation to epithelial regeneration. Nature. (2015) 519:57. 10.1038/nature14228 25731159 PMC4447318

